# HCBS CARE COORDINATION AND BACKUP CARE PLANS DURING THE COVID-19 PANDEMIC IN KANSAS

**DOI:** 10.1093/geroni/igae098.1046

**Published:** 2024-12-31

**Authors:** Tracey LaPierre, Carrie Wendel-Hummell, Jennifer Babitzke, Darcy Sullivan, Danielle Olds

**Affiliations:** University of Kansas, Lawrence, Kansas, United States; University of Kansas, Lawrence, Kansas, United States; University of Kansas, Lawrence, Kansas, United States; University of Kansas, Lawrence, Kansas, United States; University of Missouri Kansas City, Kansas City, Missouri, United States

## Abstract

Backup care plans are a requirement of Medicaid-funded Home and Community Based Services (HCBS), but research on the effectiveness of backup care plans is lacking. The COVID-19 pandemic posed a prolonged emergency situation in which HCBS recipients may have needed to utilize their backup care plans. Data about experiences with HCBS services during the pandemic in Kansas came from 100 surveys of HCBS service recipients and 70 in-depth interviews with service recipients, direct support workers, family caregivers, and providers. One-third of HCBS recipients reported not having a backup care plan. Of those that had a backup care plan, less than half agreed that their plan prepared them for the pandemic. Yet, this was a time of great need for back up care, as participants widely reported increased difficulty finding and retaining direct support workers, as well as periods when their workers or caregivers were unavailable due to quarantine and other pandemic factors. 39% of service recipients reported going without care for 2 weeks or more, largely due to not having a primary or backup worker. In interviews, study participants reported that the quality of backup care plans and care coordination declined with the introduction of managed care in Kansas, with care coordination undergoing additional strain during the pandemic. These findings point to a need to better engage HCBS stakeholders in the development of backup care plans, update them more frequently, and ensure they are appropriate for long-term emergencies.

